# Inorganic nitrate, hypoxia, and the regulation of cardiac mitochondrial respiration—probing the role of PPARα

**DOI:** 10.1096/fj.201900067R

**Published:** 2019-03-14

**Authors:** James A. Horscroft, Katie A. O’Brien, Anna D. Clark, Ross T. Lindsay, Alice Strang Steel, Nathan E. K. Procter, Jules Devaux, Michael Frenneaux, Stephen D. R. Harridge, Andrew J. Murray

**Affiliations:** * Department of Physiology, Development, and Neuroscience, University of Cambridge, Cambridge, United Kingdom;; † Centre for Human and Applied Physiological Sciences, King’s College London, London, United Kingdom; and; ‡ Bob Champion Research and Education Building, University of East Anglia, Norwich, United Kingdom

**Keywords:** heart, metabolism, mitochondria, fatty acids

## Abstract

Dietary inorganic nitrate prevents aspects of cardiac mitochondrial dysfunction induced by hypoxia, although the mechanism is not completely understood. In both heart and skeletal muscle, nitrate increases fatty acid oxidation capacity, and in the latter case, this involves up-regulation of peroxisome proliferator-activated receptor (PPAR)α expression. Here, we investigated whether dietary nitrate modifies mitochondrial function in the hypoxic heart in a PPARα-dependent manner. Wild-type (WT) mice and mice without PPARα (*Ppara*^−/−^) were given water containing 0.7 mM NaCl (control) or 0.7 mM NaNO_3_ for 35 d. After 7 d, mice were exposed to normoxia or hypoxia (10% O_2_) for the remainder of the study. Mitochondrial respiratory function and metabolism were assessed in saponin-permeabilized cardiac muscle fibers. Environmental hypoxia suppressed mass-specific mitochondrial respiration and additionally lowered the proportion of respiration supported by fatty acid oxidation by 18% (*P* < 0.001). This switch away from fatty acid oxidation was reversed by nitrate treatment in hypoxic WT but not *Ppara*^−/−^ mice, indicating a PPARα-dependent effect. Hypoxia increased hexokinase activity by 33% in all mice, whereas lactate dehydrogenase activity increased by 71% in hypoxic WT but not *Ppara*^−/−^ mice. Our findings indicate that PPARα plays a key role in mediating cardiac metabolic remodeling in response to both hypoxia and dietary nitrate supplementation.—Horscroft, J. A., O’Brien, K. A., Clark, A. D., Lindsay, R. T., Steel, A. S., Procter, N. E. K., Devaux, J., Frenneaux, M., Harridge, S. D. R., Murray, A. J. Inorganic nitrate, hypoxia, and the regulation of cardiac mitochondrial respiration—probing the role of PPARα.

The mammalian heart is often described as a metabolic omnivore, in reference to its ability to oxidize a variety of substrates in order to meet ATP demands (1). Although the healthy heart predominantly uses fatty acid oxidation (FAO) to meet these requirements under fasting conditions, under hypoxic conditions, FAO is down-regulated in favor of a relative increase in glucose metabolism (2), which requires less O_2_ per ATP synthesized (1). Key to this response is a reduction in the expression of peroxisome proliferator-activated receptor (PPAR)α, a ligand-activated transcription factor expressed in liver, heart, kidney, and to a lesser extent skeletal muscle (3). When activated, PPARα increases the expression of a number of genes involved in mitochondrial fatty acid import [*e.g.*, carnitine palmitoyltransferase 1b (*Cpt1b*)] and β-oxidation [*e.g.*, 3-hydroxyacyl dehydrogenase (*Hadh*), Acyl-CoA Dehydrogenase Medium Chain (*Acadm*), Uncoupling protein 3 (*Ucp3*)] (3). In mice, cardiac-specific ablation of the hypoxia-inducible factor (HIF)-1β, increased PPARα expression and transcriptional activity, and also increased FAO (4), suggesting that HIF signaling attenuates PPARα in hypoxia. Indeed, in cardiomyocytes, HIF-1α activation decreased PPARα DNA binding activity (5). In addition to a suppression of FAO, mitochondrial pyruvate oxidation is also suppressed in hypoxia *via* the phosphorylation of pyruvate dehydrogenase (PDH) by pyruvate dehydrogenase kinase 1 (PDK1), which is induced by HIF-1α in hypoxic cells (6, 7). Thus, mitochondrial respiration would be suppressed in favor of glycolytic ATP production.

In the hypoxic rodent heart, the transcriptional activity of PPARα is down-regulated in association with a suppression of FAO (8, 9) and an increase in glycolysis (8). As such, the cardiac metabolic phenotype of hypoxic mice resembles that of mice without PPAR receptor (*Ppara*^−/−^), and notably, no further suppression of FAO occurs in *Ppara*^−/−^ mice following exposure to hypoxia (8). Moreover, although hypoxic exposure results in an impaired cardiac energetic reserve in both humans (10) and rodents (8), increasing PPARα activity and FAO in hypoxic mice through a high-fat diet did not improve energetics and in fact worsened contractile function (8), thus it appears that down-regulation of FAO in the hypoxic heart is protective. Recent work, however, has suggested that both FAO and energetics might be preserved in hypoxic tissues by dietary supplementation with inorganic nitrate (NO_3_^−^).

Dietary inorganic NO_3_^−^ is principally acquired through the consumption of leafy, green vegetables and has effects on mitochondrial function, which may be beneficial to human health (11). NO_3_^−^ is reduced to nitrite (NO_2_^−^) *via* oral NO_3_^−^ reductase in commensal bacteria (12). NO_2_^−^ is then converted to NO in the stomach by acid disproportionation (13) and is absorbed into the bloodstream in which it can be oxidized to NO_2_^−^ by ceruloplasmin (14) or to NO_3_^−^ by hemoglobin (15). Under conditions of moderate hypoxia or acidosis or both, NO_2_^−^ may be reduced to NO by one of several NO_2_^−^ reductases, including xanthine oxidoreductase (16), deoxyhemoglobin (17), deoxymyoglobin (18), and eNOS (19). Under such conditions, endogenous NO production from l-arginine and O_2_ *via* the NOS enzymes is attenuated because of the low partial pressure of O_2_; thus, NO_3_^−^ supplementation may prevent a hypoxia-induced fall in NO bioavailability.

A major physiologic role of NO is to induce vasodilatation upon its release from the endothelium in response to a range of stimuli (20). NO binds to the heme group of soluble guanylyl cyclase inducing cGMP production (21). This in turn activates cGMP-dependent protein kinase G, which results in smooth muscle relaxation and vasodilatation *via* a reduction in intracellular [Ca^2+^] (22), thus enhancing blood flow and O_2_ delivery. Additionally, supplementation with moderate doses of dietary NO_3_^−^ partially offsets the rise in circulating erythropoietin and hemoglobin in hypoxic rats (23), which might prevent the microcirculatory dysfunction associated with an increased hematocrit (24), further improving O_2_ delivery. Indeed, native Tibetan highlanders have high levels of plasma NO_3_^−^ (25) and lower blood-hemoglobin concentrations ([Hb]_b_) than acclimatised lowlanders at any given altitude (26), and this is associated with superior forearm blood flow (25). Supplementation of dietary NO_3_^−^ under hypoxic conditions may therefore preserve O_2_ delivery to respiring tissues.

In addition to the effects on O_2_ delivery, NO regulates multiple aspects of oxidative metabolism in respiring tissues. NO induces mitochondrial biogenesis through the up-regulation of PPARγ coactivator 1α (27). Within mitochondria, NO competes with O_2_ at complex IV of the electron transfer system (ETS), leading to partial inhibition of electron transport and control over reactive oxygen species signaling (28). NO also reacts with the superoxide ion (O_2_^−^) to form peroxynitrite (29), which acts as an endogenous toxicant (30). Moreover, NO can induce a post-translational modification of complex I *via* *S*-nitrosation, resulting in its inhibition (31), which has implications both for respiratory function and reactive oxygen species production.

It has been reported that supplementation with dietary NO_3_^−^ lowers the O_2_ cost of exercise in humans (32) by increasing mitochondrial thermodynamic efficiency (11), and accordingly, NO_3_^−^ could be beneficial in hypoxia. In the hypoxic rat heart, NO_3_^−^ supplementation prevented the down-regulation of ETS complex I expression and activity and the depression of mitochondrial FAO while lowering markers of oxidative stress and protecting ATP levels (33). It is not clear, however, whether this NO_3_^−^-mediated protection resulted from a direct effect on the cardiomyocyte or through improvements in O_2_ delivery that offset the challenge of hypoxia. Notably, however, NO_3_^−^ supplementation enhanced mitochondrial FAO capacity in both the heart (33) and skeletal muscle (34) of normoxic rats. Mechanistically, NO_3_^−^ increased PPARα transcriptional activity in skeletal muscle, with no increase in FAO seen in *Ppara*^−/−^ mice (34). Moreover, a similar effect on PPARα transcription was seen in cultured myocytes under constant, well-oxygenated conditions (34), suggesting a role for NO_3_^−^ supplementation beyond any influence of NO on hemodynamics.

Dietary NO_3_^−^, therefore, protects β-oxidation in the hypoxic heart (33) and increases β-oxidation in skeletal muscle *via* PPARα activation (34). PPARα transcriptional activity is suppressed in the hypoxic rodent heart, although expression of PPARα itself may be unchanged (9). The interaction between NO_3_^−^ and PPARα in the hypoxic heart, however, remains unclear, and more specifically, it is not known whether PPARα is essential for the protective effects on mitochondrial respiratory function and FAO elicited by NO_3_^−^. We therefore investigated this in wild-type (WT) mice (*Ppara*^+/+^) and *Ppara*^−/−^ mice that were exposed to environmental hypoxia or normoxia with and without supplementation with a moderate concentration of dietary NO_3_^−^. We previously reported that NO_3_^−^ protected aspects of skeletal muscle mitochondrial respiratory function in these mice in hypoxia and found this occurred independently of PPARα (35). However, it has been suggested that in the skeletal muscle of *Ppara*^−/−^ mice, high expression of PPARβ/δ compensates for the loss of PPARα (36). Moreover, expression of PPARα is higher in heart than skeletal muscle (3). Here, we focused on the role of PPARα in NO_3_^−^-mediated effects on the hypoxic heart, hypothesizing that NO_3_^−^ regulates mitochondrial function and β-oxidation in the hypoxic heart in a manner dependent upon PPARα activity.

## MATERIALS AND METHODS

Animal work was carried out in accordance with United Kingdom Home Office regulations under the Animals in Scientific Procedures Act, and underwent review by the University of Cambridge Animal Welfare and Ethical Review Committee. Procedures involving live animals were carried out by a license holder in accordance with these regulations.

### Study design

**Figure 1:**
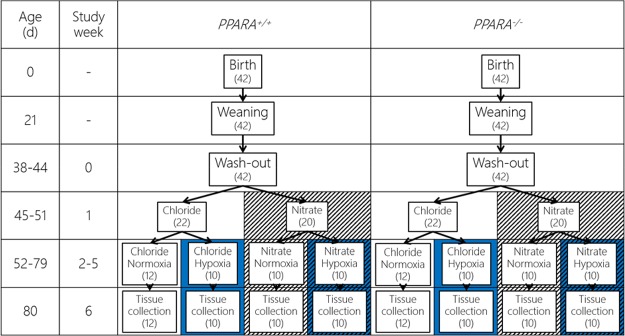
Study design. Each stage of the study took place within the ages shown ±4 d, and the length of each stage was identical for each mouse. The left-hand section represents mice with PPARα receptor (*Ppara*^+/+^), whereas the right-hand section represents mice without (*Ppara*^−/−^). The number in brackets indicates the number of mice per group. Chloride, 0.7 mM NaCl in distilled water *ad libitum*; NO_3_^−^, 0.7 mM NaNO_3_ in distilled water *ad libitum*; normoxia, 21% atmospheric O_2_; hypoxia, 10% atmospheric O_2_.

The overall study design has been previously described (35). Mice were bred on a pure 129Ev/Sv background with 10 backcrosses. The original breeding pairs of *Ppara*^+/+^ and *Ppara*^−/−^ mice were a kind gift of Frank Gonzalez [National Institutes of Health (NIH), Bethesda, MD, USA]. Mice were housed in a temperature-, humidity-, and light-controlled environment (23°C) from birth with a 12-h light/dark cycle. Normoxic mice were housed under the same environmental conditions as those in the hypoxia chamber. Mice were provided with a standard quality-controlled diet RM1(E) (65.0% carbohydrate, 13.1% crude protein, 3.5% crude fat, 10 mg/kg NO_3_^−^, trace NO_2_^−^; Special Diet Services, Essex, United Kingdom) and distilled water *ad libitum*. At 6 wk of age, mice from each genotype were randomly assigned to receive sodium chloride (0.7 mM) as a control or sodium NO_3_^−^ (0.7 mM NaNO_3_) in their drinking water. After a further 7 d, mice from each genotype/treatment combination were equally and randomly assigned to remain under normoxic conditions (21% O_2_) or transferred to hypoxic (10% O_2_) conditions in a hypoxia chamber (PFI Systems, Milton Keynes, United Kingdom). Mice were maintained under these conditions for 28 d (**Fig. 1**). Body mass, food intake, and water intake were measured weekly.

Mice were killed 80 ± 4 d after birth by dislocation of the neck. The chest cavity was opened, and the heart was removed and immediately placed in an ice-cold biopsy preservation medium, comprising 2.77 mM CaK_2_EGTA, 7.23 mM K_2_EGTA, 6.56 mM MgCl_2_·6H_2_O, 20 mM taurine, 15 mM phosphocreatine, 20 mM imidazole, 0.5 mM DTT, 50 mM 4-morpholineethanesulfonic hydrate, and 5.77 mM Na_2_ATP, pH 7.1. The heart was blotted and trimmed of extraneous tissue before being separated into 3 sections: the apex was kept in ice-cold biopsy preservation medium for high-resolution respirometry, whereas the middle section and base were snap frozen in liquid nitrogen. Meanwhile, a droplet of blood was collected from the tail vein and loaded into a microcuvette to quantify [Hb]_b_ using a HemoCue Hb 201 Analyzer (Quest Diagnostics, Angelholm, Sweden).

### High-resolution respirometry

Muscle fiber bundles were dissected from the heart and permeabilized using saponin as previously described (37). Mitochondrial respiratory function was assessed at 37°C using an Oxygraph-2K (Oroboros Instruments, Innsbruck, Austria) in respiratory medium comprising 0.5 mM EGTA, 3 mM MgCl_2_.6H_2_O, 20 mM taurine, 10 mM KH_2_PO_4_, 20 mM 4-(2-hydroxyethyl)-1-piperazineethanesulfonic acid (HEPES), 1 mg/ml bovine serum albumin, 60 mM K-lactobionate, and 110 mM sucrose, pH 7.1. Respiratory medium was hyperoxygenated at the start of each experiment and periodically throughout each assay by lifting the stopper of the oxygraph chamber slightly to introduce a gas phase; this was done before injecting pure O_2_ gas into the gas phase and then resealing the chamber once the desired O_2_ concentration was reached. O_2_ concentrations in the chambers were thus maintained between 250 and 500 µM in order to negate limitations associated with O_2_ diffusion (37); all reported respiratory fluxes were recorded within this range.

Two substrate-inhibitor titration assays were performed to investigate respiratory control of different components of the mitochondrial system. Assay 1 was based on a previously described protocol (38) with the concentrations optimized for permeabilized cardiac fibers and was designed to investigate β-oxidation of fatty acids. Assay 2 was adapted from a previously described protocol (39) and aimed to characterize control of different substrate-supported pathways over oxidative phosphorylation (oxphos).

#### Assay 1

The addition of malate (2 mM), plus substrates for CPT1, palmitoyl coenzyme A (40 μM), and carnitine (5 mM), resulted in nonphosphorylating respiration (LEAK) constrained by the activity of CPT1 (CPT1*_L_*). ADP (10 mM) resulted in oxphos also constrained by CPT1 (CPT1*_P_*). In order to bypass CPT1 and investigate β-oxidation capacity (PalM*_P_*), palmitoyl carnitine (20 μM) was added. Finally, cytochrome *c* (10 μM) was added to assess the integrity of the outer mitochondrial membrane.

#### Assay 2

Administration of octanoyl carnitine (0.2 mM) with malate (2 mM) resulted in LEAK respiration (OctM*_L_*). ADP (10 mM) addition resulted in oxphos respiration dependent on β-oxidation of medium chain fatty acids (OctM*_P_*). Pyruvate (5 mM) was then added (PM*_P_*), followed by glutamate (10 mM) to support electron flux through the N-pathway *via* complex I (GM*_P_*). Succinate (10 mM) was then administered to additionally support electron flux through the S-pathway *via* complex II (GMS*_P_*). Following this, cytochrome *c* (10 μM) was added to assess mitochondrial membrane integrity before rotenone (0.5 μM) was administered to inhibit complex I and restrict electron flux to the S-pathway *via* complex II (S*_P_*).

#### Coupling control ratios

In both assays, the oxphos coupling efficiency (*j*) (*i.e.*, the proportion of oxphos capacity that could not be explained by LEAK-limited respiration), was calculated as Eq. 1.
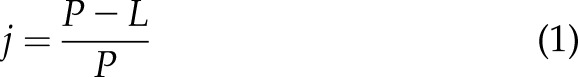
where *j* is the oxphos coupling efficiency, *L* is the LEAK respiration rate, and *P* is the oxphos respiration rate.

#### Substrate control ratios

The flux control of CPT1 over β-oxidation was assessed from assay 1 by expressing CPT1-limited oxphos as a ratio of β-oxidation-limited oxphos to give a flux control ratio (FCR) in Eq. 2:
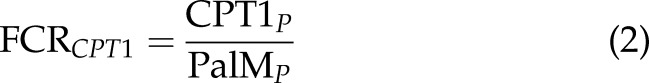
From assay 2, oxphos supported by the F-pathway (*via* β-oxidation; Eq. 3), the N-pathway (*via* complex I; Eq. 4), and the S-pathway (*via* complex II; Eq. 5) were expressed as a ratio of maximal oxphos to discern the proportion of oxygen flux controlled by these pathways as follows:
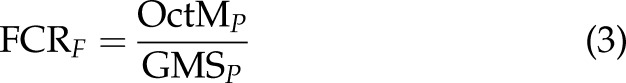

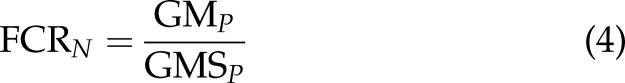

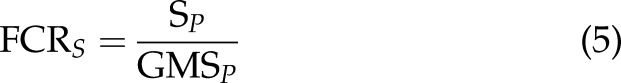
Finally, the ratio of oxphos supported by octanoyl carnitine and malate to oxphos supported by pyruvate and malate in assay 2 was used as an indicator of the relative capacity for fatty acids as a substrate for mitochondrial respiration (Eq. 6):
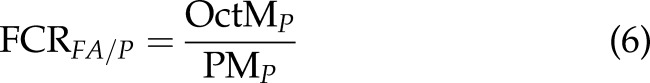


### Enzyme activity assays

Cardiac muscle homogenates were prepared from the contents of the oxygraph chamber. In brief, the entire contents of each chamber were removed, and the chambers were washed with 2 ml respiratory medium. The original contents and wash were combined with 2 μl of protease inhibitor (Complete Protease Inhibitor Cocktail; Roche, Basel, Switzerland) and 40 μl of Triton X-100 (1%). The solution was then homogenized using a Polytron (25,000 rpm, 30 s) (PT-10-35 GT; Kinematic, Lucerne, Switzerland). The homogenate was centrifuged (10,000 rpm, 10 min, 4°C) and the supernatant removed and stored at −80°C until use.

In addition, whole tissue homogenates were prepared. Approximately 10 mg of cardiac muscle from the midsection of the heart was homogenized with an Eppendorf pestle in an Eppendorf tube containing 300 μl of homogenization buffer (20 mM HEPES, 1 mM EDTA, 0.1% Triton X-100, pH 7.2). The samples were then centrifuged (1000 *g*, 30 s, 4°C) and the supernatant collected.

Protein concentration of chamber and tissue homogenates was measured using the Quick Start Bradford protein assay (Bio-Rad, Hercules, CA, USA).

Enzyme activity assays were carried out at 37°C using an Evolution 220 spectrophotometer (Thermo Fisher Scientific, Waltham, MA, USA). Cardiac citrate synthase (CS) activity was quantified using a method previously described (40) with a sample size of 10 μg of protein. The assay buffer contained 20 mM Tris, 5,5′dithiobis2-nitrobenzoicacid, and 0.3 mM acetyl CoA, pH 8.0. The reaction was initiated by the addition of 0.5 mM oxaloacetate, and an absorbance change at 412 nm was measured.

Activity of the β-oxidation enzyme HADH was measured as previously described (41) with a sample size of 20 μg of protein. The assay buffer contained 50 mM imidazole, 0.15 mM NADH, and 0.1% Triton X-100, pH 7.4. Recording of absorbance change at 340 nm was initiated 10 s after addition of 0.1 mM acetoacetyl coenzyme A.

Hexokinase activity was measured with a sample size of 60 μg of protein as previously described (42). The assay buffer contained 20 mM imidazole, 1 mM ATP, 5 mM 7H_2_O.MgCl, 5 mM DTT, 2 mM NAD^+^, and 3.125 U glucose-6-phosphate dehydrogenase, pH 7.4. Recording of absorbance change at 340 nm was initiated 10 s after addition of 5 mM glucose.

Activity of lactate dehydrogenase (LDH) was quantified essentially as previously described (41) with a sample size of 2 μg of protein. The assay buffer contained 50 mM HEPES and 0.3 mM NADH, pH 7.0. Recording of absorbance change at 340 nm was initiated 10 s after addition of pyruvate.

### Pyruvate dehydrogenase expression and phosphorylation

Samples of left ventricle that had been snap frozen in liquid nitrogen were added to 100–150 µl of NP40 cell lysis buffer (Thermo Fisher Scientific) containing Halt protease and phosphatase inhibitor cocktail (Thermo Fisher Scientific). Samples were manually crushed then homogenized on ice using a Pellet Pestle (MilliporeSigma, Burlington, MA, USA). Homogenates were then snap frozen using liquid nitrogen and allowed to thaw on ice, after which they were homogenized for a second time. Samples underwent a further 2 freeze-thaw cycles using liquid nitrogen, vortexing each time, before centrifugation at 16,700 *g* for 10 min at 4°C. Supernatants were collected and stored at −80°C. Protein concentration was determined using the Bio-Rad DC Assay (Bio-Rad). Samples were loaded into Laemmli buffer at a concentration of 1.5 mg/ml and underwent SDS-PAGE using 10% acrylamide gels under reducing conditions for ∼1 h at 0.08 A, after which they were transferred onto PVDF membrane (GE Healthcare, Waukesha, WI, USA) for ∼1.5 h at 0.38 A. Membranes were subsequently blocked for a minimum of 2 h in either 5% (w/v) bovine serum albumin or skimmed milk in Tris-buffered saline with Tween, as appropriate. Primary antibody targets were Phospho(serine 232)-PDH (1:2000; Calbiochem, San Diego, CA, USA), Phospho(serine 293)-PDH (1:1000; Abcam, Cambridge, MA, USA), Phospho(serine 300)-PDH (1:1000; Calbiochem), PDH (1:1000; Cell Signaling Technology, Danvers, MA, USA), and β-actin (1:5000; MilliporeSigma). Secondary detection was carried out using horseradish peroxidase–conjugated goat anti-rabbit (1:1000; Cell Signaling Technology) or goat anti-mouse (1:1500; Dako, Jena, Germany) antibodies. Membranes were developed using Pierce ECL Western blotting substrate (Thermo Fisher Scientific), and images were captured using a ChemiDoc-It2 imager (Ultra-Violet Products, Upland, CA, USA) with VisionWorksLS 8.1.2. software (Ultra-Violet Products). Images were analyzed using ImageJ (NIH) and normalized to β-actin expression.

### Statistics

In order to investigate the individual effects of hypoxic exposure, dietary NO_3_^−^, and *Ppara^−/−^* as well as the interactions between these effects, a 3-way ANOVA was performed. The statistical approach used has been previously described (43). Initially, the 3-way interaction was considered for significance. If there was a significant 3-way interaction, main effects and 2-way interactions were disregarded, and a *post hoc* Tukey’s honestly significant difference (HSD) test was performed. Pairwise comparisons of groups between which only one of the independent variables differed were considered, and the results were recorded. If the 3-way interaction was not significant but ≥1 2-way interactions were significant, a *post hoc* Tukey’s HSD test was performed to investigate each 2-way interaction. None of the main effects of variables involved in the significant 2-way interactions were considered, whereas the main effects of variables not involved in significant interactions were. In instances where no 3- or 2-way interactions were significant, the main effects of all 3 independent variables were considered. Main effects combine all 4 groups in each state of 1 independent variable and make a pairwise comparison between these combinations (*e.g.*, all 4 WT *vs.* all 4 *Ppara*^−/−^ groups). All analyses were carried out using R software (The R Foundation for Statistical Computing, Vienna, Austria), and values of *P* < 0.05 were considered significant.

Graphs were generated using Prism 7 software (GraphPad, La Jolla, CA, USA) and follow a color/pattern scheme whereby white indicates normoxia (21% O_2_), blue indicates hypoxia (10% O_2_), block color indicates chloride (Cl^−^), and striped indicates NO_3_^−^-treated groups. In addition, the WT groups are separated from the *Ppara^−/−^* groups. Graphs display results as means ± sem. Statistically significant differences between groups are indicated in black (differences between genotypes), blue (differences between normoxia and hypoxia), and orange (interactions linked to NO_3_^−^ treatment *vs.* chloride).

## RESULTS

### Animal data

Body mass, blood hemoglobin, and food, water, and NO_3_^−^ intake have been previously reported for these mice (35).

Briefly, food and water intake fell in hypoxic mice in wk 2 (the first week of hypoxic exposure). Food intake recovered in hypoxic animals over subsequent weeks, such that it was the same in all groups at the end of the study. Water intake recovered more quickly than food intake and was the same in all groups from wk 3 onward. Inorganic NO_3_^−^ intake calculated from food and water intake and over the course of the study was 153–257 µM/kg/d in NO_3_^−^-treated mice, compared with 18–31 µM/kg/day in chloride-treated mice.

Body mass was the same in all WT mice at the start of the study, but *Ppara*^−/−^ mice were 6% lighter than WTs. At the end of the study, *Ppara*^−/−^ mice were 5% lighter than WTs (*P* < 0.01, PPARα main effect), whereas hypoxic mice were 4% lighter than normoxic mice (*P* < 0.001, hypoxia main effect) and NO_3_^−^-treated mice were 3% lighter than chloride-treated mice (*P* < 0.05, NO_3_^−^ main effect). [Hb]_b_, which was measured at the end of the study, was higher in hypoxic mice than normoxic mice (35).

### Cardiac mitochondrial respiration

#### Mass-specific respiration

**Figure 2:**
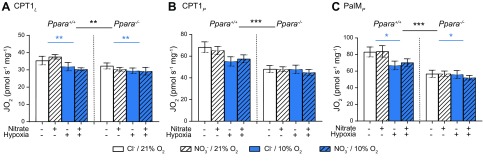
Mitochondrial respiratory function (J_O2_) from assay 1 normalized to mass. *A*) Malate and palmitoyl CoA stimulated LEAK respiration (CPT1*_L_*). *B*) CPT1-limited oxphos (CPT1*_P_*). *C*) Oxphos supported by the F-pathway *via* β-oxidation (PalM*_P_*) in permeabilized cardiac muscle fibers from *Ppara*^+/+^ and *Ppara*^−/−^ mice following normoxia (white bars, 21% O_2_) or hypoxia (blue bars, 10% O_2_) and chloride (open bars, 0.7 mM NaCl) or NO_3_^−^ (striped bars, 0.7 mM NaNO_3_) supplementation. Error bars indicate sem. Black asterisks indicate main/PPARα effect; blue asterisks indicate hypoxia effect; *n* = 8–11/group. *P < 0.05, **P < 0.01, ***P < 0.001.

Assay 1 was used to measure aspects of FAO and the control exerted by CPT1, with respiration rates initially normalized to wet weight of tissue (**Fig. 2**). Mass-specific respiration rates in *Ppara*^−/−^ mice were 10% (*P* < 0.01, Fig. 2*A*), 23% (*P* < 0.001, Fig. 2*B*), and 27% lower (*P* < 0.001, Fig. 2*C*, all PPARα main effect) when limited by proton leak (CPT1*_L_*), CPT1 flux (CPT1*_P_*), and β-oxidation (PalM*_P_*), respectively. Hypoxic exposure resulted in 11% lower LEAK respiration (*P* < 0.01, Fig. 2*A*) and 13% lower oxphos supported by β-oxidation (*P* < 0.05, Fig. 2*C*, both hypoxia main effect). Taken together, these data confirm that PPARα supports FAO in the mouse heart, whereas hypoxia suppresses the mass-specific capacity for β-oxidation.

**Figure 3:**
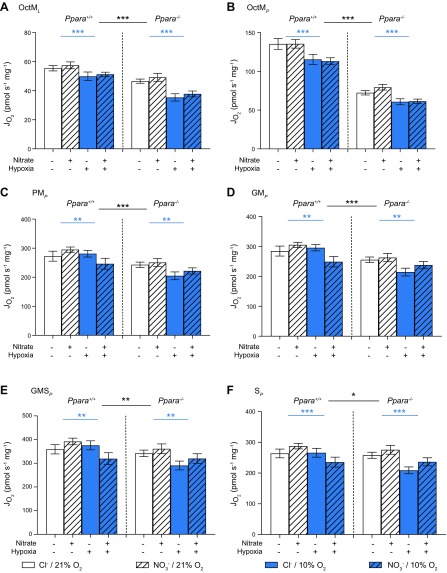
Mitochondrial respiratory function (J_O2_) from assay 2 normalized to mass. *A*) Malate and octanoyl carnitine stimulated LEAK respiration (OctM*_L_*). *B*) Oxphos supported by the F-pathway *via* β-oxidation (OctM*_P_*). *C*) Oxphos supported by pyruvate and malate through the N-pathway *via* complex I (PM*_P_*). *D*) Oxphos supported by glutamate and malate through the N-pathway *via* complex I (GM*_P_*). *E*) Oxphos supported by glutamate, malate, and succinate through the NS-pathway *via* complexes I and II (GMS*_P_*). *F*) Oxphos supported by succinate following the addition of rotenone (S*_P_*) in permeabilized cardiac muscle fibers from *Ppara*^+/+^ and *Ppara*^−/−^ mice following normoxia (white bars, 21% O_2_) or hypoxia (blue bars, 10% O_2_) and chloride (open bars, 0.7 mM NaCl) or NO_3_^−^ (striped bars, 0.7 mM NaNO_3_) supplementation. Error bars indicate sem. Black asterisks indicate main/PPARα effect; blue asterisks indicate hypoxia effect; *n* = 8–11/group. *P < 0.05, **P < 0.01, ***P < 0.001.

Assay 2 was used to measure respiration supported by FAO and substrates for the N-pathway *via* complex I and the S-pathway *via* complex II of the ETS, with respiration rates initially normalized to wet weight of tissue (**Fig. 3**). All respiration rates were lower in *Ppara*^−/−^ mice compared with wild types and were lower in hypoxic mice compared with normoxic mice, whereas dietary NO_3_^−^ supplementation had no effect on mass-specific respiration rates.

In *Ppara*^−/−^ mice, LEAK respiration (OctM*_L_*) and oxphos respiration (OctM*_P_*) supported by malate and octanoyl carnitine were 21% lower (*P* < 0.001, Fig. 3*A*) and 45% lower (*P* < 0.001, Fig. 3*B*, both PPARα main effect) than in WT mice, underlining the role of PPARα in regulating cardiac FAO. Meanwhile, oxphos supported by pyruvate and malate (PM*_P_*) was 16% lower (*P* < 0.001, Fig. 3*C*), by glutamate and malate (GM*_P_*) was 14% lower (*P* < 0.001, Fig. 3*D*), by glutamate, malate, and succinate (GMS*_P_*) was 9% lower (*P* < 0.01, Fig. 3*E*), and by succinate following the addition of rotenone (S*_P_*) was 10% lower (*P* < 0.01, all PPARα main effect, Fig. 3*F*).

In hypoxic mice, OctM*_L_* and OctM*_P_* were 16% (*P* < 0.001, Fig. 3*A*) and 17% (*P* < 0.001, Fig. 3*B*) lower than in normoxic mice, whereas PM*_P_* was 10% lower (*P* < 0.01, Fig. 3*C*), GM*_P_* was 10% lower (*P* < 0.01, Fig. 3*D*), GMS*_P_* was 10% lower (*P* < 0.01, Fig. 3*E*), and S*_P_* was 13% lower (*P* < 0.01, Fig. 3*F*, all hypoxia main effect).

Together, these data show that both *Ppara* ablation and hypoxic exposure lower oxidative capacity in the mouse heart, with suppression of fatty acid-supported respiration occurring to a greater extent than respiration supported by other substrates. This is particularly pronounced in the case of *Ppara* ablation.

#### CS-specific respiration

Both hypoxic exposure and *Ppara*^−/−^ resulted in a general lowering of mass-specific respiration rates in mouse heart. This might be attributable to a loss of cardiac mitochondrial content or to changes in respiration per mitochondrial unit, and therefore, to further probe the effects of hypoxia, NO_3_^−^, and PPARα on mitochondrial respiration, we extracted the contents of the oxygraph chambers and measured the activity of CS in chamber homogenates. CS activity is a putative marker of mitochondrial content (44), and by expressing mitochondrial respiration rates relative to CS activity, we were able to consider respiratory capacities per mitochondrial unit.

**Figure 4:**
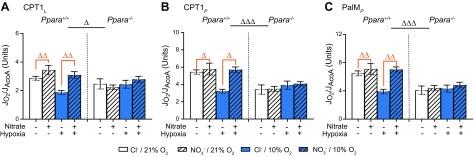
Mitochondrial respiratory function (J_O2_) from assay 1 normalized to CS activity (J_AcoA_). *A*) Malate and palmitoyl CoA stimulated LEAK respiration (CPT1*_L_*). *B*) CPT1-limited oxphos (CPT1*_P_*). *C*) Oxphos supported by the F-pathway *via* β-oxidation (PalM*_P_*) in permeabilized cardiac muscle fibers from *Ppara*^+/+^ and *Ppara*^−/−^ mice following normoxia (white bars, 21% O_2_) or hypoxia (blue bars, 10% O_2_) and chloride (open bars, 0.7 mM NaCl) or NO_3_^−^ (striped bars, 0.7 mM NaNO_3_) supplementation. Error bars indicate sem. Black Δ, 2-way interaction; orange Δ, NO_3_^−^ effect; black Δ, PPARα effect; *n* = 4–6/group. ^Δ^*P* < 0.05, ^ΔΔ^*P* < 0.01, ^ΔΔΔ^*P* < 0.001.

In assay 1, hypoxia had no effect on any CS-corrected respiration rate (**Fig. 4**). Dietary NO_3_^−^ supplementation increased CPT1*_L_* by 38% (*P* < 0.01, Fig. 4*A*), CPT1*_P_* by 33% (*P* < 0.05, Fig. 4*B*), and PalM*_P_* by 36% (*P* < 0.01, Fig. 4*C*, all Tukey’s test of NO_3_^−^/PPARα interaction) in WT but not *Ppara*^−/−^ mice. *Ppara* knockout lowered all CS-corrected respiration rates. In normoxic mice, *Ppara* knockout lowered CPT1*_L_* by 25% (*P* < 0.05, Fig. 4*A*), CPT1*_P_* by 38% (*P* < 0.001, Fig. 4*B*), and PalM*_P_* by 38% (*P* < 0.001, Fig. 4*C*, all Tukey’s test of hypoxia/PPARα interaction). In NO_3_^−^-supplemented mice, *Ppara* knockout lowered CPT1*_L_* by 23% (*P* < 0.05, Fig. 4*A*), CPT1*_P_* by 34% (*P* < 0.001, Fig. 4*B*), and PalM*_P_* by 35% (*P* < 0.001, Fig. 4*C*, all Tukey’s test of NO_3_^−^/PPARα interaction). Taken together, these data suggest that *Ppara* ablation attenuates FAO capacity per mitochondrial unit and that NO_3_^−^ increases FAO per mitochondrial unit in a PPARα-dependent manner.

**Figure 5:**
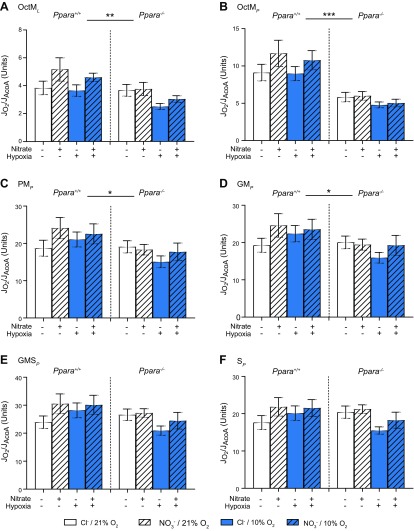
Mitochondrial respiratory function (J_O2_) from assay 2 normalized to CS activity (J_AcoA_). *A*) Malate and octanoyl carnitine stimulated LEAK respiration (OctM*_L_*). *B*) Oxphos supported by the F-pathway *via* β-oxidation (OctM*_P_*). *C*) Oxphos supported by pyruvate and malate through the N-pathway *via* complex I (PM*_P_*). *D*) Oxphos supported by glutamate and malate through the N-pathway *via* complex I (GM*_P_*). *E*) Oxphos supported by glutamate, malate, and succinate through the NS-pathway *via* complexes I and II (GMS*_P_*). *F*) Oxphos supported by by succinate following the addition of rotenone (S*_P_*) in permeabilized cardiac muscle fibers from *Ppara*^+/+^ and *Ppara*^−/−^ mice following normoxia (white bars, 21% O_2_) or hypoxia (blue bars, 10% O_2_) and chloride (open bars, 0.7 mM NaCl) or NO_3_^−^ (striped bars, 0.7 mM NaNO_3_) supplementation. Error bars indicate sem. Black asterisks indicate PPARα effect; *n* = 4–6/group. *P < 0.05, **P < 0.01, ***P < 0.001.

In Assay 2, neither NO_3_^−^ supplementation nor hypoxic treatment affected CS-corrected respiration rates (**Fig. 5**). *Ppara* ablation, however, lowered CS-corrected OctM*_L_* by 25% (*P* < 0.01, Fig. 5*A*), OctM*_P_* by 27% (*P* < 0.001, Fig. 5*B*), PM*_P_* by 19% (*P* < 0.05, Fig. 5*C*), and GM*_P_* by 17% (*P* < 0.05, Fig. 5*D*, all PPARα main effect). GMS*_P_* and S*_P_* were unaffected by NO_3_^−^, hypoxia, or genotype (Fig. 5*E, F*). These data further support the notion that *Ppara* ablation attenuates cardiac mitochondrial FAO capacity, in addition to any effect on mitochondrial content.

#### Oxphos coupling efficiency

Oxphos coupling efficiency, indicating the increase in respiration following addition of ADP relative to the resulting respiration rate, was calculated for both assays (Supplemental Fig. S1). In assays 1 and 2, oxphos coupling efficiency was 19 and 29% lower, respectively, in *Ppara*^−/−^ mice compared with WT mice (*P* < 0.001, PPARα main effect). Neither NO_3_^−^ supplementation nor hypoxic exposure affected oxphos coupling efficiency. These data suggest that although PPARα enhanced fatty acid-supported respiration in both LEAK and oxphos states, the effect on the oxphos state was proportionally greater.

#### Substrate control ratios

**Figure 6:**
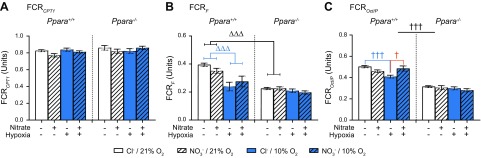
Substrate control ratios. Ratios indicate CPT1 control over β-oxidation (FCR*_CPT1_*) (*A*); the contribution of the F-pathway *via* β-oxidation to maximal oxphos (FCR*_F_*) (*B*), and the capacity for oxphos supported by octanoyl carnitine plus malate relative to pyruvate plus malate (FCR*_Oct/P_*) (*C*) in permeabilized cardiac muscle fibers from *Ppara*^+/+^ and *Ppara*^−/−^ mice, following normoxia (white bars, 21% O_2_) or hypoxia (blue bars, 10% O_2_) and chloride (open bars, 0.7 mM NaCl) or NO_3_^−^ (striped bars, 0.7 mM NaNO_3_) supplementation. Error bars indicate sem. Two-way interaction (Δ); 3-way interaction (†); NO_3_^−^ effect (orange †); hypoxia effect (blue Δ, †); PPARα effect (black Δ, †); *n* = 8–10/group. ^†^*P* < 0.05, ^ΔΔΔ^*P* < 0.001, ^†††^*P* < 0.001.

To further investigate the effects of NO_3_^−^ supplementation, hypoxic exposure, and PPARα on cardiac mitochondrial respiration and the interactions between these 3 factors, we used substrate control ratios to interrogate control points and substrate-led pathways (**Fig. 6**).

Firstly, to understand the effect of these factors on the activity of CPT1 (an enzyme responsible for the entry of long chain fatty acid substrates into mitochondria *via* the addition of carnitine), we expressed CPT1*_P_* relative to PalM*_P_* (FCR*_CPT1_*, Fig. 6*A*). Here, we found that the substrate control ratio was unaffected by any factor. This indicates that although oxphos supported by CPT1 substrates and oxphos supported by β-oxidation are suppressed in *Ppara*^−/−^ mice and hypoxic mice, the effects on these 2 respiration rates are proportionally similar.

Next, we sought to understand the contribution of 3 substrate-led pathways to total oxphos capacity, expressing oxphos supported by: *1*) the F-pathway *via* β-oxidation (OctM*_P_*), *2*) the N-pathway *via* complex I (GM*_P_*), and *3*) the S-pathway *via* complex II (S*_P_*), relative to maximal oxphos (GMS_P_).

In WT, but not *Ppara*^−/−^ mice, exposure to hypoxia lowered oxphos respiration through the F-pathway relative to maximal oxphos (FCR*_F_*) by 32% (*P* < 0.001, Tukey’s test of hypoxia/PPARα interaction, Fig. 6*B*). Similarly, this was 41% lower in normoxic *Ppara*^−/−^ mice compared with WT mice. This suggests that hypoxic exposure lowers FAO relative to maximal oxphos in a PPARα-dependent manner.

Oxphos supported by the N-pathway *via* complex I, relative to maximal oxphos (FCR*_N_*), was 7% lower in *Ppara*^−/−^ mice compared with WT mice (*P* < 0.001, PPARα main effect, Supplemental Fig. S2*A*) but was unaffected by hypoxic exposure or NO_3_^−^ supplementation. Meanwhile, oxphos supported by the S-pathway *via* complex II (FCR*_S_*) was unaffected by *Ppara* ablation or hypoxia but was 2% higher in NO_3_^−^-supplemented mice (*P* < 0.05, NO_3_^−^ main effect, Supplemental Fig. S2*B*).

Finally, we expressed octanoyl carnitine-supported oxphos (OctM*_P_*) relative to pyruvate-supported oxphos (PM*_P_*) in order to indicate the relative capacity for FAO compared with pyruvate-linked respiration through the N-pathway (FCR*_Oct/P_*, Fig. 6*C*). The relative capacity for FAO was decreased by hypoxia in chloride-supplemented WT mice by 18% (*P* < 0.001, Tukey’s test of NO_3_^−^/hypoxic/PPARα interaction) but not in NO_3_^−^-supplemented WT mice nor in either group of hypoxic *Ppara*^−/−^ mice. Dietary NO_3_^−^ reversed this effect in hypoxic WT mice, increasing the relative capacity for FAO by 17% (*P* < 0.05, Tukey’s test of NO_3_^−^/hypoxia/PPARα). Again, no effect was seen in *Ppara*^−/−^ mice. The relative capacity for FAO was, however, lower in *Ppara*^−/−^ mice (*P* < 0.001, Tukey’s test of NO_3_^−^/hypoxia/PPARα interaction). These data therefore demonstrate that in mouse heart: *1*) *Ppara* ablation induces a substrate switch away from fatty acids, *2*) exposure to hypoxia induces a substrate switch away from fatty acids acting *via* decreased PPARα activity, and *3*) dietary NO_3_^−^ prevents the hypoxia-induced substrate switch in a PPARα-dependent manner.

### Enzyme activities

**Figure 7:**
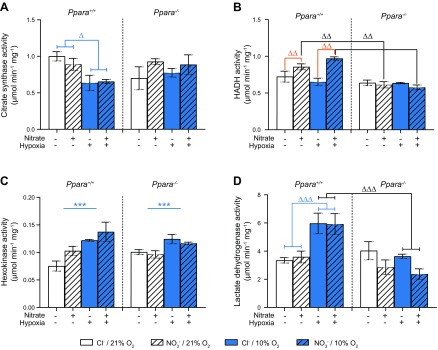
Enzyme activities in mouse heart tissue homogenates. Maximal activity of CS (*A*), HADH (*B*), hexokinase (*C*), and LDH (*D*) from *Ppara*^+/+^ and *Ppara*^−/−^ mice, following normoxia (white bars, 21% O_2_) or hypoxia (blue bars, 10% O_2_) and chloride (open bars, 0.7 mM NaCl) or NO_3_^−^ (striped bars, 0.7 mM NaNO_3_) supplementation. Error bars indicate sem. Symbols in brackets denote significance of a test of a combination of groups (*i.e.*, main effects or 2-way interactions); *n* = 4–5/group. ***P < 0.001 (hypoxia main effect), ^ΔΔ^*P* < 0.01 (NO_3_^−^ effect following NO_3_^−^/PPARα interaction), ^ΔΔΔ^*P* < 0.001 (hypoxia effect following hypoxia/PPARα interaction), ^ΔΔ^*P* < 0.01 (PPARα effect following NO_3_^−^/PPARα interaction), ^ΔΔΔ^*P* < 0.01, (PPARα effect following hypoxia/PPARα interaction).

Hypoxic exposure resulted in 32% lower CS activities in WT mouse hearts compared with those of their normoxic counterparts (*P* < 0.05, Tukey’s test of hypoxia/PPARα interaction), but it did not affect CS activity in *Ppara*^−/−^ mice (**Fig. 7*A***).

NO_3_^−^-supplemented WT mice had 33% higher activities of the β-oxidation enzyme HADH in heart (*P* < 0.01), whereas no effect was seen in *Ppara*^−/−^ mice (Fig. 7*B*). Moreover, in *Ppara*^−/−^ mice supplemented with NO_3_^−^, but not by chloride, HADH activity was 35% lower than in WT counterparts (*P* < 0.01, Tukey’s test of NO_3_^−^/PPARα interaction). This suggests that dietary NO_3_^−^ increases HADH activity in cardiac tissue *via* a PPARα-dependent mechanism.

Hexokinase activity was 33% higher in hypoxic groups relative to normoxic counterparts (*P* < 0.001, hypoxia main effect), with no significant effect on hexokinase activity resulting from NO_3_^−^ supplementation or genotype (Fig. 7*C*). Hypoxia also increased LDH activity by 71% in WT but not *Ppara*^−/−^ mice (*P* < 0.001, Tukey’s test of hypoxia/PPARα interaction, Fig. 7*D*). Hypoxia was also permissive of a PPARα effect because, under hypoxic but not normoxic conditions, *Ppara* ablation resulted in 50% lower LDH activities (*P* < 0.001, Tukey’s test of hypoxia/PPARα interaction). Dietary NO_3_^−^ did not affect the activity of either hexokinase or LDH. Thus, hypoxia increased hexokinase activity in a manner that was independent of dietary NO_3_^−^ and PPARα but increased LDH in a manner that was dependent upon PPARα.

### Pyruvate dehydrogenase levels and phosphorylation

**Figure 8:**
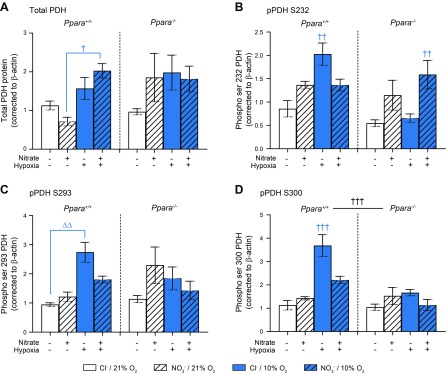
Total PDH levels and phosphorylation. *A*) Protein levels of total PDH. *B–D*) Phosphorylation of PDH at E1α site serine 232 (pPDH S232) (*B*), serine 293 (pPDH S293) (*C*), and serine 300 (pPDH S300) (*D*) in *Ppara*^+/+^ and *Ppara*^−/−^ mice, following normoxia (white bars, 21% O_2_) or hypoxia (blue bars, 10% O_2_) and chloride (open bars, 0.7 mM NaCl) or NO_3_^−^ (striped bars, 0.7 mM NaNO_3_) supplementation. Error bars indicate sem. Two-way interaction (Δ); 3-way interaction (†); hypoxia effect (blue Δ, †); PPARα effect (black Δ, †); *n* = 8–10/group. ^†^*P* < 0.05, ^ΔΔ^*P* < 0.01; ^††^*P* < 0.01, ^†††^*P* < 0.001.

To examine the regulation of pyruvate oxidation, total and phosphorylated PDH levels were measured in cardiac homogenates by immunoblotting. There was no specific effect of hypoxia on total PDH levels (**Fig. 8*A***), although total levels were higher in hypoxic NO_3_^−^-supplemented WT mice compared with their normoxic counterparts (*P* < 0.05, Tukey’s test of hypoxia/ NO_3_^−^/PPARα interaction). Hypoxia increased phosphorylation of PDH at serine residues 232, 293, and 300 in WT mice, and this was prevented by NO_3_^−^ supplementation (*P* < 0.01, Tukey’s test of hypoxia/ NO_3_^−^/PPARα interaction, Fig. 8*B–D*). There was no clear effect of hypoxia on phosphorylation of any serine residue in *Ppara*^−/−^ mice. At serine 232, NO_3_^−^ supplementation appeared to enhance phosphorylation in hypoxic *Ppara*^−/−^ mice (*P* < 0.05, Tukey’s test of hypoxia/ NO_3_^−^/PPARα interaction, Fig. 8*B*). Overall, these data suggest hypoxia increases PDH inhibition by phosphorylation in a PPARα-dependent manner, whereas NO_3_^−^ prevents this.

## DISCUSSION

We have previously demonstrated that a moderate dose of dietary NO_3_^−^ protects mitochondrial respiratory function, FAO, and energetics in the hypoxic rat heart (33) and enhances skeletal muscle FAO capacity through a mechanism dependent upon activation of PPARα (34). Decreased PPARα transcriptional activity appears to be a key aspect of the cardiac metabolic response to hypoxia (8, 9), so here we sought to understand whether PPARα plays a role in mediating the protective effect of NO_3_^−^ in the hypoxic heart.

We found that hypoxic exposure was associated with the suppression of mass-specific respiratory capacity and CS activity in mouse heart, with no protective effect of NO_3_^−^. Mass-specific respiration was also lower in *Ppara*^−/−^ mice compared with WT mice. Hypoxia increased phosphorylation of PDH in WT mice and also increased the capacity for glycolysis with increased hexokinase activity in all mice and increased LDH activity in WT mice. In addition to the general suppression of oxidative capacity, hypoxia resulted in a particular down-regulation of FAO capacity in WT mice. This was reversed by NO_3_^−^ treatment, an effect not apparent in *Ppara*^−/−^ mice. Similarly, NO_3_^−^ increased activity of HADH in WT mice but not *Ppara*^−/−^ mice.

Strengths of this study include the use of high-resolution measurements of oxygen flux to consider multiple aspects of FAO capacity with both CPT1-dependent and independent substrates. Our inclusion of both mass-specific and mitochondrial-specific FAO capacity measurements as well as FAO in proportion to maximal oxphos capacity is also a strength. It should be noted, however, that all measurements were carried out under conditions in which oxygen and substrates were saturating, and although our data indicate alterations in respiratory capacity, there may be further, subtle differences in mitochondrial respiration *in vivo* at physiologic oxygen and substrate concentrations that we have not measured here. The use of permeabilized fibers, rather than isolated mitochondria, allowed us to measure respiration in the entire population of cardiac mitochondria, albeit without allowing us to distinguish between effects on the interfibrillar and subsarcolemmal populations of mitochondria (45). A further strength of this study was the statistical approach, which, although complex, allowed us to directly address the key hypothesis that NO_3_^−^ exerts effects in hypoxia acting through PPARα. The 3-way ANOVA approach allowed us to test the interaction between these 3 factors and represented a conservative approach, eliminating the need for multiple comparisons and strengthening the conclusions of this study. This approach, however, does increase the chance of type 2 errors and is not suitable for testing differences between just 2 of the groups [*e.g.*, the effect of hypoxia on WT (non-NO_3_^−^-supplemented) mice], for which a more targeted study design would be more appropriate.

The use of mice, rather than rats, is both a strength and a weakness of this study. The *Ppara*^−/−^ mouse has been studied extensively, particularly in relation to the role of PPARα in regulating FAO in various tissues, and was a valuable component of this study. Mice, however, differ from rats and humans in their NO production rates (46) and concentrations of circulating NO_3_^−^/ NO_2_^−^ levels (47). Moreover, we only used male mice in this study, which may be a limitation because, for example, gender dimorphic differences in the antiplatelet response to NO_3_^−^ supplementation have been observed in humans (48). As with our previous studies, we were able to precisely control NO_3_^−^ intake using a standardized quality-controlled diet and deionized water (23, 33–35, 49, 50). Although the anorexic effects in the early stages of hypoxic exposure may have confounded our findings, the long duration of the hypoxic exposure proved to be a strength because water intake and therefore NO_3_^−^ intake stabilized in hypoxic mice by wk 2, matching that of their normoxic counterparts for the remainder of the study.

Unlike our previous findings in rat heart (33), however, we saw no protective effect of NO_3_^−^ supplementation on mass-specific oxphos. This could be due to species differences, indeed 17 mo of NaNO_3_ supplementation at 1 mM did not alter plasma NO_3_^−^ concentration in mice (51), whereas 0.7 mM NaNO_3_ increased circulating NO_3_^−^ in rats (33). It should be noted, however, that both the duration (4 wk) and degree of hypoxia (10% O_2_) were more severe in this study than in our previous work (2 wk at 13% O_2_), and this may have limited the effectiveness of NO_3_^−^ supplementation. Of note, the increased activity of LDH is an established hypoxia response in the heart (52), and this was seen in both chloride-supplemented and NO_3_^−^-supplemented mice, perhaps suggesting that NO_3_^−^ supplementation did not fully restore O_2_ delivery to these hearts.

The general suppression of mass-specific respiratory capacity, particularly in the context of more prolonged and severe hypoxia, might be explained by a loss of mitochondrial content in these hearts. Indeed, we saw that hypoxia lowered CS activity in the hearts of WT mice in this study, whereas there was no such change in CS in our previous study that employed a milder hypoxia protocol (33). In skeletal muscle, the suppression of CS is dependent on both duration and degree of hypoxia (53). CS activity is a putative marker of mitochondrial density, correlating with mitochondrial volume in the skeletal muscle of healthy, young adult humans (44), although whether this correlation holds in the mouse heart under all conditions described here is unknown.

In addition to the general effect of hypoxia on mass-specific respiration, a particular suppression of FAO was seen. This was indicated by a fall in CS-corrected respiration rates for fatty acid substrates and also by substrate control ratios expressing FAO as a proportion of maximal oxphos or in relation to pyruvate-supported oxphos. This effect of hypoxia was seen in the hearts of WT but not *Ppara*^−/−^ mice, indicating that the hypoxic suppression of FAO is primarily driven by decreased PPARα activity, as suggested previously (8). There was no specific effect on CPT1-supported FAO (with palmitoyl CoA plus malate) compared with CPT1-independent respiration (with palmitoyl carnitine plus malate), indicating that although CPT1 activity may be down-regulated, there are also effects of a similar magnitude downstream, probably on β-oxidation capacity, although in agreement with our previous work (9) HADH activity was unaltered by hypoxia. Although the severity of the hypoxic stimulus used here may have prevented any restorative effect of NO_3_^−^ on mass-specific respiration, NO_3_^−^ did prevent the particular suppression of FAO, but only in WT mice, suggesting that PPARα is necessary for these NO_3_^−^-mediated effects to occur. Moreover, NO_3_^−^ supplementation increased HADH activity in the hearts of WT mice but not *Ppara*^−/−^ mice.

In addition to the effect of hypoxia, mass-specific respiratory capacity was lower in the hearts of *Ppara*^−/−^ mice than those of WT mice, and as might be expected, this was most pronounced for respiration rates supported by fatty acid substrates, although a suppression of respiration supported by substrates for the N-pathway *via* complex I (pyruvate, glutamate, and malate) and the S-pathway *via* complex II (succinate) was also seen. When corrected for CS activity, respiration supported by fatty acid substrates remained lower in *Ppara*^−/−^ mice compared with WT mice, underlining its importance in regulating cardiac FAO, but of note, respiration supported by N-pathway substrates was also lower in *Ppara*^−/−^ mice. Unlike in WT mice, there was no effect of NO_3_^−^ supplementation on FAO capacity or HADH activity in *Ppara*^−/−^ mice, demonstrating that PPARα is an essential mediator of the effects of NO_3_^−^ on cardiac metabolism in hypoxia, which is in line with our initial hypothesis.

Activities of hexokinase and LDH were increased following hypoxia as did PDH phosphorylation, all suggesting an up-regulation of glycolytic capacity. Although the increase in hexokinase activity was not dependent on PPARα, occurring in mice of both genotypes, the increase in LDH dehydrogenase activity only occurred in WT mice, suggesting an unexpected influence of PPARα on *Ldh* expression. PPARβ/δ has been shown to decrease the expression ratio of *Ldha*/*Ldhb* in skeletal muscle (54), but we are not aware of any previously reported effects of PPARα. Whereas *Ldhb* encodes an LDH isoform that encourages the conversion of lactate to pyruvate, the protein product of *Ldha* favors the conversion of pyruvate to lactate. It is plausible, therefore, that PPARα ablation is compensated by an increase in PPARβ/δ, which prevents a hypoxia-induced increase in *Ldha*/*Ldhb*, though this would have to be confirmed with gene expression studies. Alternatively, PPARα is known to increase PDK4 activity, leading to an attenuation of PDH activity. This could increase pyruvate concentration, which may feed forward to increase LDH activity. Indeed, whereas hypoxia increased PDH phosphorylation in WT mice, potentially *via* HIF-dependent up-regulation of PDK1 (6, 7), this effect was blunted in *Ppara*^−/−^ mice. Taken together, these data implicate a switch away from oxidative metabolism toward glycolysis in the hypoxic mouse heart.

Our data therefore suggest that PPARα plays a key role in regulating the process of metabolic remodeling that occurs in the hypoxic heart and that PPARα is essential for the effects of dietary NO_3_^−^ in preserving oxidative metabolism. This is in contrast to our previous findings in skeletal muscle in which the metabolic response to hypoxia ^+/−^ NO_3_^−^ was found to be the same in WT mice and *Ppara*^−/−^ mice (35). The difference between the 2 tissues, and the greater dependence on PPARα in the heart, might be explained by the higher expression of PPARα in cardiac muscle compared with skeletal muscle (3). NO_3_^−^ supplementation did exert effects in hypoxic skeletal muscle, but this may have been due to effects *via* PPARβ/δ (36) or through hemodynamic changes that alter blood flow and O_2_ delivery (20). Our data do not definitively address whether NO_3_^−^ exerts its effects by altering blood flow and therefore O_2_ delivery or through direct effects on tissue metabolism. Although PPARα is an essential effector in this response, its transcriptional activity may simply be modified by HIF signaling in response to changes in tissue oxygenation in order to ensure that oxidative metabolism is supported only when sufficient oxygen is available. Arguing against this is our finding here that hexokinase and LDH activity were increased by hypoxia in both chloride-supplemented and NO_3_^−^-supplemented hearts, indicating that although the dose of NO_3_^−^ employed here did modulate FAO, it was not sufficient to ameliorate the fall in oxygenation through improved blood flow. Moreover, our previous work in cultured myotubes indicated that NO_3_^−^ enhanced PPARα activity without changes in oxygenation (34), and it would be interesting to replicate this in cardiomyocytes. It seems likely that NO_3_^−^ exerts complementary but separate effects on blood flow and metabolism, ensuring that tissue O_2_ supply and demand are matched.

Our work has implications for a number of conditions in which cardiac and skeletal muscle metabolism is pathologically altered, particularly where derangements in NO production or availability have been implicated. For instance, eNOS knockout mice develop a metabolic syndrome-like phenotype, including obesity and insulin resistance, yet features of the pathology were reversed following NO_3_^−^ supplementation (55). It has been suggested that NO_3_^−^ supplementation may be of benefit to patients with heart failure with reduced ejection fraction in whom eNOS activity is impaired (56). A recent study demonstrated improvements in NO bioavailability and muscle power following NO_3_^−^ supplementation in 9 patients with heart failure with reduced ejection fraction (56), and it would be interesting to see if this was associated with changes in tissue metabolism in addition to improvements in blood flow. The failing heart itself is energy-starved (57) and characterized by a down-regulation of FAO (1, 57), which may improve the efficiency of O_2_ utilization. It was shown, however, that oxygenation was not impaired in the nonischemic failing myocardium (58), and thus therapeutic strategies that enhance oxidative metabolism, including perhaps NO_3_^−^ supplementation, may be of benefit to these patients.

In summary, hypoxia suppresses oxidative metabolism in the heart and enhances glycolytic capacity. In addition, there is a particular suppression of FAO in the hypoxic heart that is mediated through decreased PPARα transcriptional activity and that can be reversed by supplementation with dietary NO_3_^−^ in a PPARα-dependent manner.

## Supplementary Material

This article includes supplemental data. Please visit *http://www.fasebj.org* to obtain this information.

## Abbreviations

CPT: carnitine palmitoyltransferase
CS: citrate synthase
ETS: electron transfer system
FAO: fatty acid oxidation
HADH: 3-hydroxyacyl dehydrogenase
[Hb]_b_: blood-hemoglobin concentration
HEPES: 4-(2-hydroxyethyl)-1-piperazineethanesulfonic acid
HIF: hypoxia-inducible factor
LEAK: nonphosphorylating respiratory leak
LDH: lactate dehydrogenase
NO_2_^−^: nitrite
NO_3_^−^: nitrate
O_2_^−^: superoxide
OctM*_L_*: LEAK respiration
OctM*_P_*: oxphos respiration
oxphos: oxidative phosphorylation
*Ppara*^-/-^: mice without PPARα
*Ppara*^+/+^: mice with PPARα
PDH: pyruvate dehydrogenase
PDK: pyruvate dehydrogenase kinase
PPAR: peroxisome proliferator-activated receptor
WT: wild type

## References

[1] Lopaschuk G. D., Ussher J. R., Folmes C. D., Jaswal J. S., Stanley W. C. (2010) Myocardial fatty acid metabolism in health and disease. Physiol. Rev. 90, 207–258 20086077 10.1152/physrev.00015.2009

[2] Essop M. F. (2007) Cardiac metabolic adaptations in response to chronic hypoxia. J. Physiol. 584, 715–726 17761770 10.1113/jphysiol.2007.143511 PMC2276994

[3] Rakhshandehroo M., Knoch B., Müller M., Kersten S. (2010) Peroxisome proliferator-activated receptor alpha target genes. PPAR Res. 2010, 612089 20936127 10.1155/2010/612089 PMC2948931

[4] Wu R., Chang H. C., Khechaduri A., Chawla K., Tran M., Chai X., Wagg C., Ghanefar M., Jiang X., Bayeva M., Gonzalez F., Lopaschuk G., Ardehali H. (2014) Cardiac-specific ablation of ARNT leads to lipotoxicity and cardiomyopathy. J. Clin. Invest. 124, 4795–4806 25329697 10.1172/JCI76737 PMC4347233

[5] Belanger A. J., Luo Z., Vincent K. A., Akita G. Y., Cheng S. H., Gregory R. J., Jiang C. (2007) Hypoxia-inducible factor 1 mediates hypoxia-induced cardiomyocyte lipid accumulation by reducing the DNA binding activity of peroxisome proliferator-activated receptor alpha/retinoid X receptor. Biochem. Biophys. Res. Commun. 364, 567–572 17963722 10.1016/j.bbrc.2007.10.062

[6] Kim J. W., Tchernyshyov I., Semenza G. L., Dang C. V. (2006) HIF-1-mediated expression of pyruvate dehydrogenase kinase: a metabolic switch required for cellular adaptation to hypoxia. Cell Metab. 3, 177–185 16517405 10.1016/j.cmet.2006.02.002

[7] Papandreou I., Cairns R. A., Fontana L., Lim A. L., Denko N. C. (2006) HIF-1 mediates adaptation to hypoxia by actively downregulating mitochondrial oxygen consumption. Cell Metab. 3, 187–197 16517406 10.1016/j.cmet.2006.01.012

[8] Cole M. A., Abd Jamil A. H., Heather L. C., Murray A. J., Sutton E. R., Slingo M., Sebag-Montefiore L., Tan S. C., Aksentijević D., Gildea O. S., Stuckey D. J., Yeoh K. K., Carr C. A., Evans R. D., Aasum E., Schofield C. J., Ratcliffe P. J., Neubauer S., Robbins P. A., Clarke K. (2016) On the pivotal role of PPARα in adaptation of the heart to hypoxia and why fat in the diet increases hypoxic injury. FASEB J. 30, 2684–2697 27103577 10.1096/fj.201500094R PMC5072355

[9] Horscroft J. A., Burgess S. L., Hu Y., Murray A. J. (2015) Altered oxygen utilisation in rat left ventricle and soleus after 14 days, but not 2 days, of environmental hypoxia. PLoS One 10, e0138564 26390043 10.1371/journal.pone.0138564 PMC4577132

[10] Holloway C. J., Montgomery H. E., Murray A. J., Cochlin L. E., Codreanu I., Hopwood N., Johnson A. W., Rider O. J., Levett D. Z., Tyler D. J., Francis J. M., Neubauer S., Grocott M. P., Clarke K.; Caudwell Xtreme Everest Research Group (2011) Cardiac response to hypobaric hypoxia: persistent changes in cardiac mass, function, and energy metabolism after a trek to Mt. Everest base camp. FASEB J. 25, 792–796 20978235 10.1096/fj.10-172999

[11] Larsen F. J., Schiffer T. A., Borniquel S., Sahlin K., Ekblom B., Lundberg J. O., Weitzberg E. (2011) Dietary inorganic nitrate improves mitochondrial efficiency in humans. Cell Metab. 13, 149–159 21284982 10.1016/j.cmet.2011.01.004

[12] Duncan C., Dougall H., Johnston P., Green S., Brogan R., Leifert C., Smith L., Golden M., Benjamin N. (1995) Chemical generation of nitric oxide in the mouth from the enterosalivary circulation of dietary nitrate. Nat. Med. 1, 546–551 7585121 10.1038/nm0695-546

[13] Benjamin N., O’Driscoll F., Dougall H., Duncan C., Smith L., Golden M., McKenzie H. (1994) Stomach NO synthesis. Nature 368, 502 10.1038/368502a0 8139683

[14] Shiva S., Wang X., Ringwood L. A., Xu X., Yuditskaya S., Annavajjhala V., Miyajima H., Hogg N., Harris Z. L., Gladwin M. T. (2006) Ceruloplasmin is a NO oxidase and nitrite synthase that determines endocrine NO homeostasis. Nat. Chem. Biol. 2, 486–493 16906150 10.1038/nchembio813

[15] Gardner P. R. (2005) Nitric oxide dioxygenase function and mechanism of flavohemoglobin, hemoglobin, myoglobin and their associated reductases. J. Inorg. Biochem. 99, 247–266 15598505 10.1016/j.jinorgbio.2004.10.003

[16] Millar T. M., Stevens C. R., Benjamin N., Eisenthal R., Harrison R., Blake D. R. (1998) Xanthine oxidoreductase catalyses the reduction of nitrates and nitrite to nitric oxide under hypoxic conditions. FEBS Lett. 427, 225–228 9607316 10.1016/s0014-5793(98)00430-x

[17] Cosby K., Partovi K. S., Crawford J. H., Patel R. P., Reiter C. D., Martyr S., Yang B. K., Waclawiw M. A., Zalos G., Xu X., Huang K. T., Shields H., Kim-Shapiro D. B., Schechter A. N., Cannon R. O. III, Gladwin M. T. (2003) Nitrite reduction to nitric oxide by deoxyhemoglobin vasodilates the human circulation. Nat. Med. 9, 1498–1505 14595407 10.1038/nm954

[18] Shiva S., Huang Z., Grubina R., Sun J., Ringwood L. A., MacArthur P. H., Xu X., Murphy E., Darley-Usmar V. M., Gladwin M. T. (2007) Deoxymyoglobin is a nitrite reductase that generates nitric oxide and regulates mitochondrial respiration. Circ. Res. 100, 654–661 17293481 10.1161/01.RES.0000260171.52224.6b

[19] Gautier C., van Faassen E., Mikula I., Martasek P., Slama-Schwok A. (2006) Endothelial nitric oxide synthase reduces nitrite anions to NO under anoxia. Biochem. Biophys. Res. Commun. 341, 816–821 16442076 10.1016/j.bbrc.2006.01.031

[20] Palmer R. M., Ferrige A. G., Moncada S. (1987) Nitric oxide release accounts for the biological activity of endothelium-derived relaxing factor. Nature 327, 524–526 3495737 10.1038/327524a0

[21] Mittal C. K., Murad F. (1977) Properties and oxidative regulation of guanylate cyclase. J. Cyclic Nucleotide Res. 3, 381–391 24062

[22] Taylor D. A., Bowman B. F., Stull J. T. (1989) Cytoplasmic Ca2+ is a primary determinant for myosin phosphorylation in smooth muscle cells. J. Biol. Chem. 264, 6207–6213 2539375

[23] Ashmore T., Fernandez B. O., Evans C. E., Huang Y., Branco-Price C., Griffin J. L., Johnson R. S., Feelisch M., Murray A. J. (2015) Suppression of erythropoiesis by dietary nitrate. FASEB J. 29, 1102–1112 25422368 10.1096/fj.14-263004 PMC4422362

[24] Martin D. S., Ince C., Goedhart P., Levett D. Z., Grocott M. P.; Caudwell Xtreme Everest Research Group (2009) Abnormal blood flow in the sublingual microcirculation at high altitude. Eur. J. Appl. Physiol. 106, 473–478 19333616 10.1007/s00421-009-1023-8 PMC2688617

[25] Erzurum S. C., Ghosh S., Janocha A. J., Xu W., Bauer S., Bryan N. S., Tejero J., Hemann C., Hille R., Stuehr D. J., Feelisch M., Beall C. M. (2007) Higher blood flow and circulating NO products offset high-altitude hypoxia among Tibetans. Proc. Natl. Acad. Sci. USA 104, 17593–17598 17971439 10.1073/pnas.0707462104 PMC2077056

[26] Beall C. M., Cavalleri G. L., Deng L., Elston R. C., Gao Y., Knight J., Li C., Li J. C., Liang Y., McCormack M., Montgomery H. E., Pan H., Robbins P. A., Shianna K. V., Tam S. C., Tsering N., Veeramah K. R., Wang W., Wangdui P., Weale M. E., Xu Y., Xu Z., Yang L., Zaman M. J., Zeng C., Zhang L., Zhang X., Zhaxi P., Zheng Y. T. (2010) Natural selection on EPAS1 (HIF2alpha) associated with low hemoglobin concentration in Tibetan highlanders. Proc. Natl. Acad. Sci. USA 107, 11459–11464 20534544 10.1073/pnas.1002443107 PMC2895075

[27] Nisoli E., Tonello C., Cardile A., Cozzi V., Bracale R., Tedesco L., Falcone S., Valerio A., Cantoni O., Clementi E., Moncada S., Carruba M. O. (2005) Calorie restriction promotes mitochondrial biogenesis by inducing the expression of eNOS. Science 310, 314–317 16224023 10.1126/science.1117728

[28] Brown G. C., Cooper C. E. (1994) Nanomolar concentrations of nitric oxide reversibly inhibit synaptosomal respiration by competing with oxygen at cytochrome oxidase. FEBS Lett. 356, 295–298 7805858 10.1016/0014-5793(94)01290-3

[29] Huie R. E., Padmaja S. (1993) The reaction of no with superoxide. Free Radic. Res. Commun. 18, 195–199 8396550 10.3109/10715769309145868

[30] Radi R. (2013) Peroxynitrite, a stealthy biological oxidant. J. Biol. Chem. 288, 26464–26472 23861390 10.1074/jbc.R113.472936 PMC3772193

[31] Clementi E., Brown G. C., Feelisch M., Moncada S. (1998) Persistent inhibition of cell respiration by nitric oxide: crucial role of S-nitrosylation of mitochondrial complex I and protective action of glutathione. Proc. Natl. Acad. Sci. USA 95, 7631–7636 9636201 10.1073/pnas.95.13.7631 PMC22706

[32] Larsen F. J., Weitzberg E., Lundberg J. O., Ekblom B. (2010) Dietary nitrate reduces maximal oxygen consumption while maintaining work performance in maximal exercise. Free Radic. Biol. Med. 48, 342–347 19913611 10.1016/j.freeradbiomed.2009.11.006

[33] Ashmore T., Fernandez B. O., Branco-Price C., West J. A., Cowburn A. S., Heather L. C., Griffin J. L., Johnson R. S., Feelisch M., Murray A. J. (2014) Dietary nitrate increases arginine availability and protects mitochondrial complex I and energetics in the hypoxic rat heart. J. Physiol. 592, 4715–4731 25172947 10.1113/jphysiol.2014.275263 PMC4253472

[34] Ashmore T., Roberts L. D., Morash A. J., Kotwica A. O., Finnerty J., West J. A., Murfitt S. A., Fernandez B. O., Branco C., Cowburn A. S., Clarke K., Johnson R. S., Feelisch M., Griffin J. L., Murray A. J. (2015) Nitrate enhances skeletal muscle fatty acid oxidation via a nitric oxide-cGMP-PPAR-mediated mechanism. BMC Biol. 13, 110 26694920 10.1186/s12915-015-0221-6 PMC4688964

[35] O’Brien K. A., Horscroft J. A., Devaux J., Lindsay R. T., Steel A. S., Clark A. D., Philp A., Harridge S. D. R., Murray A. J. (2018) PPARα-independent effects of nitrate supplementation on skeletal muscle metabolism in hypoxia. [E-pub ahead of print] Biochim. Biophys. Acta. Mol. Basis Dis. 10.1016/j.bbadis.2018.07.027 PMC6414754 30055294

[36] Muoio D. M., MacLean P. S., Lang D. B., Li S., Houmard J. A., Way J. M., Winegar D. A., Corton J. C., Dohm G. L., Kraus W. E. (2002) Fatty acid homeostasis and induction of lipid regulatory genes in skeletal muscles of peroxisome proliferator-activated receptor (PPAR) alpha knock-out mice. Evidence for compensatory regulation by PPAR delta. J. Biol. Chem. 277, 26089–26097 12118038 10.1074/jbc.M203997200

[37] Pesta D., Gnaiger E. (2012) High-resolution respirometry: OXPHOS protocols for human cells and permeabilized fibers from small biopsies of human muscle. Methods Mol. Biol. 810, 25–58 22057559 10.1007/978-1-61779-382-0_3

[38] Heather L. C., Cole M. A., Tan J. J., Ambrose L. J., Pope S., Abd-Jamil A. H., Carter E. E., Dodd M. S., Yeoh K. K., Schofield C. J., Clarke K. (2012) Metabolic adaptation to chronic hypoxia in cardiac mitochondria. Basic Res. Cardiol. 107, 268 22538979 10.1007/s00395-012-0268-2

[39] Horscroft J. A., Kotwica A. O., Laner V., West J. A., Hennis P. J., Levett D. Z. H., Howard D. J., Fernandez B. O., Burgess S. L., Ament Z., Gilbert-Kawai E. T., Vercueil A., Landis B. D., Mitchell K., Mythen M. G., Branco C., Johnson R. S., Feelisch M., Montgomery H. E., Griffin J. L., Grocott M. P. W., Gnaiger E., Martin D. S., Murray A. J. (2017) Metabolic basis to Sherpa altitude adaptation. Proc. Natl. Acad. Sci. USA 114, 6382–6387 28533386 10.1073/pnas.1700527114 PMC5474778

[40] Houle-Leroy P., Garland T. Jr., Swallow J. G., Guderley H. (2000) Effects of voluntary activity and genetic selection on muscle metabolic capacities in house mice Mus domesticus. J. Appl. Physiol. (1985) 89, 1608–1616 11007602 10.1152/jappl.2000.89.4.1608

[41] McClelland G. B., Dalziel A. C., Fragoso N. M., Moyes C. D. (2005) Muscle remodeling in relation to blood supply: implications for seasonal changes in mitochondrial enzymes. J. Exp. Biol. 208, 515–522 15671340 10.1242/jeb.01423

[42] Levett D. Z., Radford E. J., Menassa D. A., Graber E. F., Morash A. J., Hoppeler H., Clarke K., Martin D. S., Ferguson-Smith A. C., Montgomery H. E., Grocott M. P., Murray A. J.; Caudwell Xtreme Everest Research Group (2012) Acclimatization of skeletal muscle mitochondria to high-altitude hypoxia during an ascent of Everest. FASEB J. 26, 1431–1441 22186874 10.1096/fj.11-197772

[43] Cohen B. H. (2013) Explaining Psychological Statistics, Wiley, Hoboken, NJ, USA

[44] Larsen S., Nielsen J., Hansen C. N., Nielsen L. B., Wibrand F., Stride N., Schroder H. D., Boushel R., Helge J. W., Dela F., Hey-Mogensen M. (2012) Biomarkers of mitochondrial content in skeletal muscle of healthy young human subjects. J. Physiol. 590, 3349–3360 22586215 10.1113/jphysiol.2012.230185 PMC3459047

[45] Palmer J. W., Tandler B., Hoppel C. L. (1985) Biochemical differences between subsarcolemmal and interfibrillar mitochondria from rat cardiac muscle: effects of procedural manipulations. Arch. Biochem. Biophys. 236, 691–702 2982322 10.1016/0003-9861(85)90675-7

[46] Siervo M., Stephan B. C., Feelisch M., Bluck L. J. (2011) Measurement of in vivo nitric oxide synthesis in humans using stable isotopic methods: a systematic review. Free Radic. Biol. Med. 51, 795–804 21672626 10.1016/j.freeradbiomed.2011.05.032

[47] Pannala A. S., Mani A. R., Spencer J. P., Skinner V., Bruckdorfer K. R., Moore K. P., Rice-Evans C. A. (2003) The effect of dietary nitrate on salivary, plasma, and urinary nitrate metabolism in humans. Free Radic. Biol. Med. 34, 576–584 12614846 10.1016/s0891-5849(02)01353-9

[48] Velmurugan S., Kapil V., Ghosh S. M., Davies S., McKnight A., Aboud Z., Khambata R. S., Webb A. J., Poole A., Ahluwalia A. (2013) Antiplatelet effects of dietary nitrate in healthy volunteers: involvement of cGMP and influence of sex. Free Radic. Biol. Med. 65, 1521–1532; erratum: 84, 385 23806384 10.1016/j.freeradbiomed.2013.06.031 PMC3878381

[49] Roberts L. D., Ashmore T., Kotwica A. O., Murfitt S. A., Fernandez B. O., Feelisch M., Murray A. J., Griffin J. L. (2015) Inorganic nitrate promotes the browning of white adipose tissue through the nitrate-nitrite-nitric oxide pathway. Diabetes 64, 471–484 25249574 10.2337/db14-0496 PMC4351918

[50] Roberts L. D., Ashmore T., McNally B. D., Murfitt S. A., Fernandez B. O., Feelisch M., Lindsay R., Siervo M., Williams E. A., Murray A. J., Griffin J. L. (2017) Inorganic nitrate mimics exercise-stimulated muscular fiber-type switching and myokine and γ-aminobutyric acid release. Diabetes 66, 674–688 28028076 10.2337/db16-0843

[51] Hezel M. P., Liu M., Schiffer T. A., Larsen F. J., Checa A., Wheelock C. E., Carlström M., Lundberg J. O., Weitzberg E. (2015) Effects of long-term dietary nitrate supplementation in mice. Redox Biol. 5, 234–242 26068891 10.1016/j.redox.2015.05.004 PMC4475696

[52] Selmeci L., Farkas A., Pósch E., Szelényi I., Sós J. (1967) The effect of hypoxia on the lactic dehydrogenase (LDH) activity of serum and heart muscle of rats. Life Sci. 6, 649–653 6040169 10.1016/0024-3205(67)90102-6

[53] Horscroft J. A., Murray A. J. (2014) Skeletal muscle energy metabolism in environmental hypoxia: climbing towards consensus. Extrem. Physiol. Med. 3, 19 25473486 10.1186/2046-7648-3-19 PMC4253994

[54] Gan Z., Burkart-Hartman E. M., Han D. H., Finck B., Leone T. C., Smith E. Y., Ayala J. E., Holloszy J., Kelly D. P. (2011) The nuclear receptor PPARβ/δ programs muscle glucose metabolism in cooperation with AMPK and MEF2. Genes Dev. 25, 2619–2630 22135324 10.1101/gad.178434.111 PMC3248683

[55] Carlström M., Larsen F. J., Nyström T., Hezel M., Borniquel S., Weitzberg E., Lundberg J. O. (2010) Dietary inorganic nitrate reverses features of metabolic syndrome in endothelial nitric oxide synthase-deficient mice. Proc. Natl. Acad. Sci. USA 107, 17716–17720 20876122 10.1073/pnas.1008872107 PMC2955084

[56] Mulkareddy V., Racette S. B., Coggan A. R., Peterson L. R. (2018) Dietary nitrate’s effects on exercise performance in heart failure with reduced ejection fraction (HFrEF). [E-pub ahead of print] Biochim. Biophys. Acta. Mol. Basis Dis. 10.1016/j.bbadis.2018.09.026 PMC6401215 30261290

[57] Neubauer S. (2007) The failing heart--an engine out of fuel. N. Engl. J. Med. 356, 1140–1151 17360992 10.1056/NEJMra063052

[58] Dass S., Holloway C. J., Cochlin L. E., Rider O. J., Mahmod M., Robson M., Sever E., Clarke K., Watkins H., Ashrafian H., Karamitsos T. D., Neubauer S. (2015) No evidence of myocardial oxygen deprivation in nonischemic heart failure. *Circ*. *Heart Fail* 8, 1088–1093 26333351 10.1161/CIRCHEARTFAILURE.114.002169 PMC4645953

