# Supplementary material for: Inorganic nitrate, hypoxia, and the regulation of cardiac mitochondrial respiration—probing the role of PPARα
Source: FASEB J. 2019 Mar 14;33(6):7563–77. doi: 10.1096/fj.201900067R (PMC6529343; doi:10.1096/fj.201900067R)
Supplement: Supplementary file 1 [file fj.201900067R.sf1.pdf]

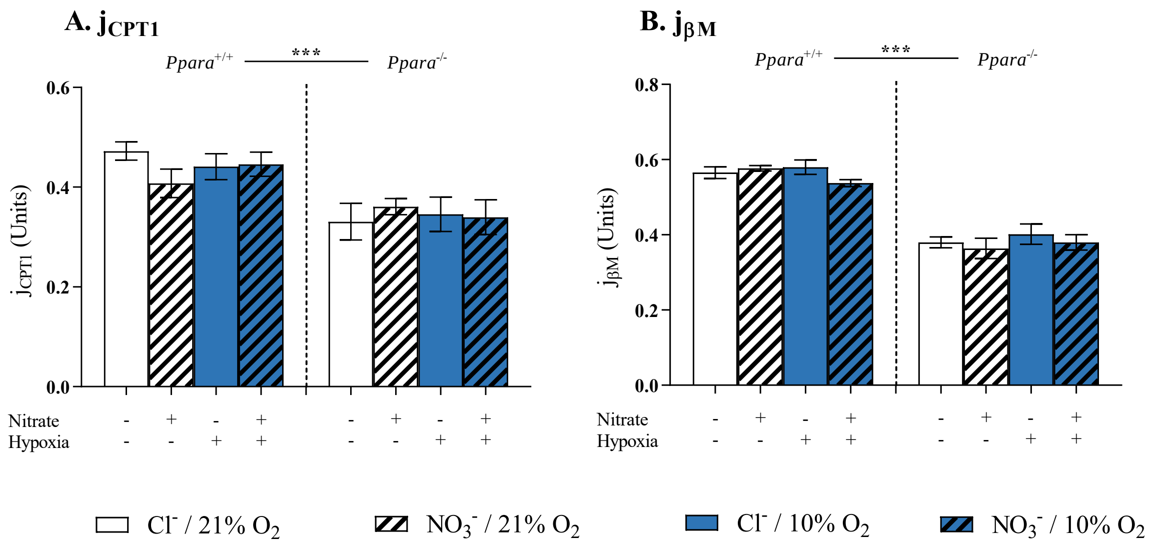

Supplementary Figure 1: OXPHOS coupling efficiencies (j)

A) From Assay 1 (j_{CPT1}) and B) Assay 2 (j_{OctM}) in permeabilised cardiac fibres from wild-type (*Ppara*$^{+/+}$) and *Ppara*$^{-/-}$ mice, following normoxia (white bars, 21% O_2) or hypoxia (blue bars, 10% O_2), and chloride (open bars, 0.7 mM NaCl) or nitrate (striped bars, 0.7 mM $NaNO_3$) supplementation. Error bars indicate SEM. *** = PPARα main effect at $P < 0.001$. n = 8-10 per group.
