# Supplementary material for: Inorganic nitrate, hypoxia, and the regulation of cardiac mitochondrial respiration—probing the role of PPARα
Source: FASEB J. 2019 Mar 14;33(6):7563–77. doi: 10.1096/fj.201900067R (PMC6529343; doi:10.1096/fj.201900067R)
Supplement: Supplementary file 2 [file fj.201900067R.sf2.pdf]

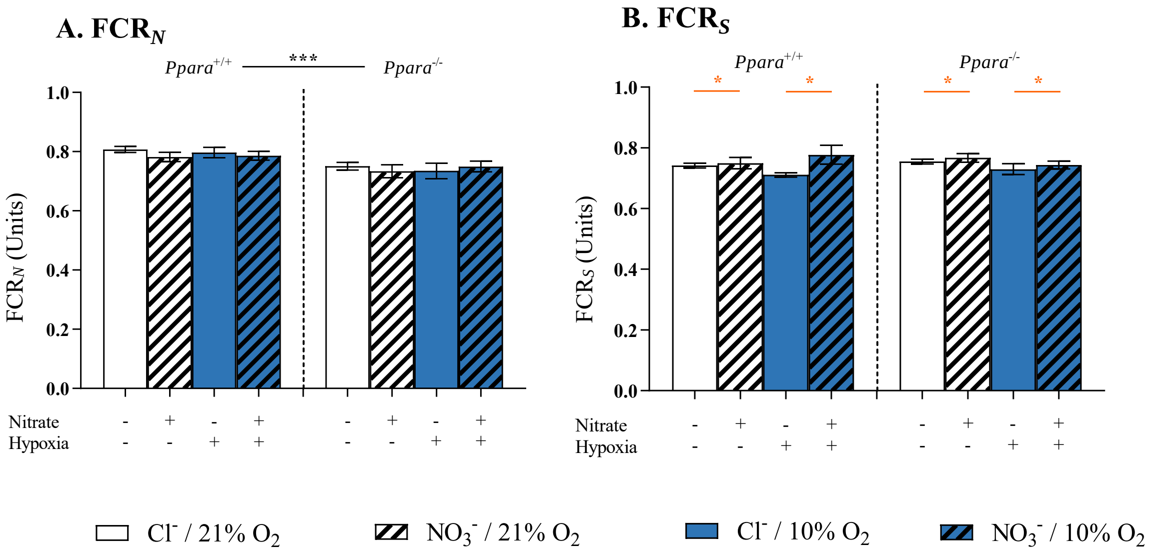

Supplementary Figure 2: Substrate control ratios

Ratios indicate the contributions of A) the N-pathway via complex I to maximal OXPHOS (FCR_N) and B) the S-pathway via complex II to maximal OXPHOS (FCR_S) in permeabilised cardiac muscle fibres from wild-type (*Ppara*$^{+/+}$) and *Ppara*$^{-/-}$ mice, following normoxia (white bars, 21% O_2) or hypoxia (blue bars, 10% O_2), and chloride (open bars, 0.7 mM NaCl) or nitrate (striped bars, 0.7 mM $NaNO_3$) supplementation. Error bars indicate SEM. * = main effect. Orange (*) symbols = nitrate effect; black (*) symbols = PPARα effect. 1 (*) symbol = $p < 0.05$; 3 (***) symbols = $p < 0.001$. Symbols in brackets (e.g. $^{(*)}$) denote significance of a test of a combination of groups (i.e. main effects or two-way interactions) as described in the text. n = 8-10 per group.
